# Development of Aptamers for RNase Inactivation in Xtract-Free™ Sample Collection and Transport Medium

**DOI:** 10.3390/diagnostics14121207

**Published:** 2024-06-07

**Authors:** Luke T. Daum, John D. Rodriguez, James P. Chambers

**Affiliations:** 1 LuJo BioScience Laboratory, San Antonio, TX 78209, USA; luke@lujobioscience.com; 2 Department of Biology, University of Texas at San Antonio, San Antonio, TX 78249, USA; jchamber@utsa.edu

**Keywords:** Xtract-Fee™ medium, sample collection, qPCR, diagnostics, extraction-free detection, point of care, home-collect, self-collect, DNA aptamers, RNase, nucleases, third generation specimen collection

## Abstract

There is a significant need to develop new environmentally friendly, extraction-free sample collection mediums that can effectively preserve and protect genetic material for point-of-care and/or self-collection, home-collection, and mail-back testing. Systematic evolution of ligands by exponential enrichment (SELEX) was used to create anti-ribonuclease (RNase) deoxyribonucleic acid (DNA) aptamers against purified RNase A conjugated to paramagnetic carboxylated beads. Following eight rounds of SELEX carried out under various stringency conditions, e.g., selection using Xtract-Free™ (XF) specimen collection medium and elevated ambient temperature of 28 °C, a panel of five aptamers was chosen following bioinformatic analysis using next-generation sequencing. The efficacy of aptamer inactivation of RNase was assessed by monitoring ribonucleic acid (RNA) integrity via fluorometric and real-time RT-PCR analysis. Inclusion of aptamers in reaction incubations resulted in an 8800- to 11,200-fold reduction in RNase activity, i.e., digestion of viral RNA compared to control. Thus, anti-RNase aptamers integrated into XF collection medium as well as other commercial reagents and kits have great potential for ensuring quality intact RNA for subsequent genomic analyses.

## 1. Introduction

Aptamers are short nucleic acid oligonucleotides (typically 20–100 bases) that, like antibodies, recognize and bind to biomolecular targets, including proteins [1,2,3,4,5,6]. They are generated through a cyclic enrichment process known as systematic evolution of ligands by exponential enrichment (SELEX) [1]. During SELEX, a large chemically synthesized library of random nucleic acid sequences (approximately 10^15^) is passed over an immobilized target molecule. Sequences that do not bind to the target are washed away, while those that do bind are subsequently teased off and amplified using polymerase chain reaction (PCR). Through repeated cycles of incubation with the target, washing, and amplification, a subset of binding sequences with high target affinity is selected, i.e., enriched [1,2,3,4,5,6].

Aptamers offer several advantages over traditional antibodies. First, they can be produced in vitro relatively easily and inexpensively in a standard molecular laboratory without the need for animals or culturing techniques. Since they are oligonucleotides, they are safe, chemically stable, and can be modified with functional groups or reporter molecules. Additionally, aptamers are small (approximately 20 kDa), which enables them to bind to molecular locations that are inaccessible to antibodies due to their larger size (around 150 kDa). Consequently, aptamers have been used for a variety of applications, including biosensing, diagnostics, therapeutics, and basic research [3,7,8,9,10]. However, to the best of our knowledge, aptamers have not been used for preservation of ribonucleic acid (RNA) genomic targets in diagnostic sample collection media as described.

Nucleases are enzymes that cleave phosphodiester bonds in nucleic acids. RNases are one of many nucleases found in various microbes and cell types across all organisms, including prokaryotes and eukaryotes [11,12,13,14,15]. They are widespread and pose a significant challenge to those laboratories that work with small quantities of RNA, especially from collected samples. Ribonucleases (RNases) can be introduced inadvertently as contaminants or, more likely, released endogenously from cells and microbes in medium used in sample collection and transport. RNase digestion in collected samples becomes even more problematic when biological specimens or environmental samples containing high levels of nucleases are collected at points-of-care, i.e., home collection or remote testing sites, pharmacies, or physician’s offices, where specimens often remain at ambient or higher temperatures until processed in the molecular diagnostic testing laboratory [16,17,18]. Thus, there is an urgent need to protect collected RNA samples from degradation by RNases, as well as degradation from the use of harsh chemicals that are dangerous to humans and the environment.

During routine respiratory collection, nasopharyngeal or nasal anterior swabs are often placed into viral or universal transport mediums (VTM and UTM). VTM and UTM are complex mixtures of sugars, salts, and buffers developed more than four decades ago for viral and bacterial culturing [19,20]. A major drawback of VTM and UTM is that they do not include any RNase-inactivating capacity. Thus, labile viral and host RNA from samples collected in VTM and UTM are susceptible to significant RNase degradation, i.e., decrease in nucleic acid targets. More recently, molecular transport mediums (MTMs) such as PrimeStore (Longhorn Vaccines and Diagnostics, Bethesda, MD, USA) and eNat (Copan Diagnostics, Brescia, Italy) were developed to disrupt cell membranes and inactivate/denature proteins including RNases [21,22,23]. MTMs are similar in composition to standard cell lysis buffer and use the same chemical approach to shear and disrupt microbial lipid bilayers [21]. However, the use of MTMs for sample collection presents four significant limitations: (1) Typically, MTMs contain toxic guanidine compounds that are extremely harmful if accidentally contacted via the skin or ingested [24,25]; (2) they are hazardous to the environment, and can release potentially toxic cyanide gas if contact with bleach products during cleanup occurs [26,27]; (3) they cannot be used for lateral flow, rapid antigen and protein detection tests; and (4) they require nucleic acid extraction prior to detection, a process that is laborious and costly, requiring additional nucleic acid extraction reagents.

Xtract-Free™ (XF) is a ‘next-generation’ biospecimen collection, storage, and transport medium recently developed for direct, extraction-free (extraction-less) use with quantitative PCR (qPCR) and other nucleic acid-based detection formats [28]. XF is an optimized, non-toxic blend of reagents that preserves RNA, DNA, and proteins of collected samples at ambient temperature and above. The reagents in XF function to gently lyse membranes, including those of viruses, and subsequently preserve labile RNA. Importantly, samples collected in XF can be analyzed directly using qPCR and non-qPCR methodologies without nucleic acid extraction [28]. Furthermore, XF contains no alcohol, guanidine, or other hazardous surfactants, making it ideal for use with lateral flow formats for at-home and self-testing use.

In this study, we report the generation of anti-RNase DNA aptamers for preservation of collected biospecimen RNA in concert with Xtract-Free™ collection medium. Our results demonstrate successful RNase inactivation by RNase-binding aptamers and their utility in a novel specimen collection medium. This is an important step for enhancement of RNA quality, impacting detection/quantitation of specific targets of interest and downstream RNA molecular analyses from collection biospecimens.

## 2. Materials and Methods

### 2.1. Conjugation of RNase A to Carboxy Paramagnetic Beads

Sera-Mag™ carboxylate-modified magnetic particles (Cytiva, Little Chalfont, UK) were used for conjugation of purified RNase A (Roche Diagnostics, Mannheim, Germany) by covalent linkage, as described below. Carboxy beads stored at 5 °C were allowed to come to ambient temperature and were mixed thoroughly prior to use. A total of 1 mL of beads (50 mg/mL) was added to 7.5 mL of nuclease-free water and mixed by pipetting. The bead suspension was magnetically partitioned with a magnet stand for 1 min until clear, after which the fluid was aspirated and discarded. Using nuclease-free water, the beads were washed two times to remove trace bead solution, followed by the addition of 7.5 mL of 0.1 M NaOH two times by mixing, magnetic partitioning for 1 min, aspiration, and discarding the fluid. Then, the beads were washed an additional two times with 7.5 mL of nuclease-free water to remove trace NaOH. A total of 7.5 mL of 0.1 M 2-(*N*-Morpholino) ethanesulfonic acid (MES; Sigma, St. Louis, MO, USA) was then added, the bead suspension was removed from the stand, and the contents were thoroughly mixed. In a sterile 15-milliliter Falcon tube, 200 mg of 1-ethyl-3-(3-dimethylaminopropyl)carbodiimide (EDC; Sigma, St. Louis, MO, USA) and 100 mg of *N*-hydroxysuccinimide (NHS) were combined with 2 mL of MES (0.1 M; pH 5) and mixed thoroughly. The carboxy bead suspension was partitioned magnetically, and the MES buffer was aspirated and discarded. To activate the carboxy beads, 2 mL of EDC/NHS solution was added and the bead suspension was vortexed briefly, and placed on a mini-tube rotator for 1 h. Following mixing, the bead suspension was placed on the magnet stand for 1 min. The partitioned solution was aspirated, and the beads were resuspended in 1 mL of MES (0.1 M; pH 5). In a separate sterile microcentrifuge tube, a solution of RNase A (3 mg/mL; Roche, Mannheim, Germany) was prepared by dilution in MES buffer (0.1 M, pH 5.0). To bind RNase to the beads, 1 mL of RNase protein (3 mg/mL) was added and mixed gently on a mini tube rotator for 1 h. Then, the reaction mixture was placed on the magnet stand, and the magnetically partitioned fluid was removed and discarded. The bead-RNase conjugate was resuspended in 2 mL of Trizma-HCl (0.1 M; Sigma, St. Louis, MO, USA) and mixed thoroughly for 1 h on a mini tube rotator. Then, the suspension was placed on the magnet stand for 1 min, and the fluid was removed and discarded. The bead-RNase conjugate was washed twice using 7.5 mL of PBS/0.1% (*v*/*v*) Tween-20 (Sigma; St. Louis, MO, USA), and finally resuspended in 7.5 mL of PBS/0.1% Tween-20, mixed well, and stored at 5 °C until used. 

### 2.2. In Vitro SELEX Selection of Anti-RNase A Aptamers

The synthetic aptamer library was synthesized by Integrated DNA Technologies (Coralville, IA, USA) and consisted of the following 86 nucleotides: 5′-TAG-GGA-ACA-GAA-GGA-CAT-ATG-AT-(*N*40)-TTG-ACT-AGT-ACA-TGA-CCA-CTT-GA-3′. A 40-nucleotide randomized region (10^25^ sequences) was flanked on both sides by a 23-nucleotide forward and reverse primer region for polymerase chain reaction (PCR). Additionally, SELEX-Forward, 5′-TAG-GGA-AGA-GAA-GGA-CAT-ATG-AT, SELEX-Reverse, 5′-TCA-AGT-GGT-CAT-GTA-CTA-GTC-AA-3′, and a 5′-phosphorylated reverse, 5′-Phos-TCA-AGT-GGT-CAT-GTA-CTA-GTC-AA-3′, were utilized (IDT, Coralville, IA, USA). The aptamer library and all primers were diluted to 100 µM stocks. All the primers were utilized at a 20 µM working dilution.

**Figure 1:**
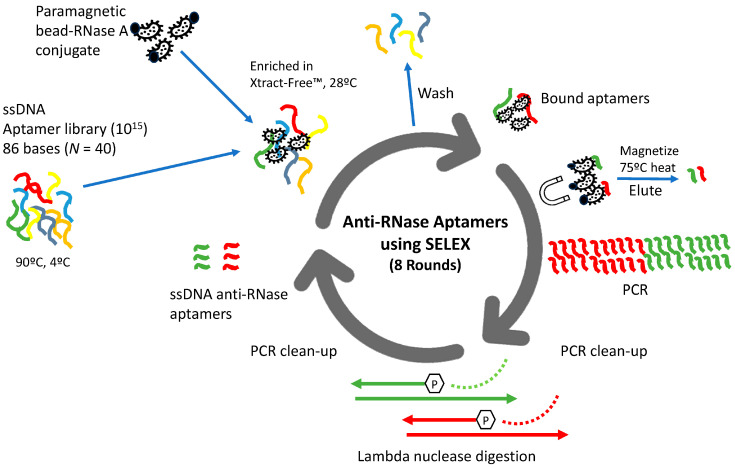
Overview of anti-RNase A aptamer production. Aptamer enrichment was performed using purified RNase A conjugated to paramagnetic carboxy beads that were incubated in Xtract-Free™ collection medium (LuJo Bioscience Laboratory, San Antonio, TX, USA) at an elevated (28 °C) temperature. The colors represent the approximately 10^15^ different species of aptamers in the original SELEX pool.

A total of 8 SELEX rounds were performed (Figure 1). A high SELEX stringency for aptamers was employed for RNase binding, since selection was performed as follows: (1) directly in Xtract-Free™ medium; and (2) at elevated ambient temperature (28 °C). For SELEX round 1, an aliquot of 100 µM aptamer library was diluted with nuclease-free water to a concentration of 10 nMol. The library was subsequently diluted to 2 nMol (10^15^ molecules) by adding 20 µL of ssDNA library to 80 µL of Xtract-Free™ medium in a 0.2 PCR reaction tube. For proper folding, the aptamers were first heated to 90 °C (10 min) and then cooled to 4 °C (15 min), using an ABI 2720PCR thermocycler (Applied BioSystems, Foster City, CA, USA). Using a magnet stand, 100 µL of bead-RNase A conjugate was washed three times with 400 µL of SELEX binding buffer and resuspended in 100 µL of Xtract-Free™ collection medium. Next, 100 µL of aptamer library was added to the tube containing 100 µL of bead-RNase conjugate/Xtract-Free™ medium. The contents were pipetted up and down several times to mix, with subsequent incubation in a heat block at elevated ambient temperature (28 °C) for 30 min with intermittent pipetting every 5 min. The mixture was magnetically partitioned and washed twice with 400 µL of Xtract-Free™ to remove unbound aptamers. During each washing step, the tube was rotated 3–5 times manually to ensure bound aptamers were not inadvertently dislodged from the beads. After two washes, bead-RNase conjugate with bound aptamers (with no fluid) was briefly removed from the stand to allow beads to settle to the bottom of the 1.5 mL tube before being placed back on the magnet stand; any remaining residual volume was carefully removed. The tube with bound aptamers was left open on the stand and air-dried for 5 min. To elute the bound aptamers, 200 µL of nuclease-free water (Thermo Fisher, Waltham, MA, USA) was added to the dried bead tube, pipetted up and down, and incubated at 70 °C for 7 min. After incubation, the microcentrifuge was briefly vortexed, placed on the magnet stand and magnetically partitioned, and the solution containing eluted aptamers was transferred to a new microcentrifuge tube. 

### 2.3. Polymerase Chain Reaction Amplification

PCR to amplify the bound aptamers was performed during each SELEX round in a 50 µL total reaction volume of “MasterMix” consisting of the following: 32 µL or nuclease-free water (Thermo Fisher, Waltham, MA, USA), 10 µL of 5X PCR buffer (Bioline, London, UK), 2 µL of forward and phosphate-labeled reverse primers (20 µM each), 1 µL of MyTaq polymerase (BioLine, London, UK), and 5 µL of eluted aptamer. The PCR reaction was performed in 0.2 mL PCR tubes, and consisted of an initial denaturation at 95 °C for 2 min, followed by 8 cycles of 94 °C for 30 s, 55 °C for 20 s, and 72 °C for 30 s. A final extension step was performed at 72 °C for 5 min. Positive and negative (no template) control reactions were included in each PCR run. A total of 10 µL of PCR product consisting of approximately 86 bp was visualized with UV illumination on a 2% agarose gel with ethidium bromide staining. Prior to visualization, the gels were electrophoresed at 90 volts for 1 h. The remaining 40 µL of PCR product was cleaned using the NEB Monarch PCR and DNA Cleanup Kit (NEB, Ipswich, MA, USA), as specified in the user’s manual.

### 2.4. Lambda Nuclease Digestion

To recover single-stranded DNA (ssDNA) for seven subsequent SELEX rounds, purified PCR amplicons were subjected to lambda nuclease digestion (NEB, Ipswich, MA, USA). Briefly, 40 µL of purified PCR product were added to 5 µL of 10X Lambda exonuclease buffer, 1 µL of enzyme, and 4 µL of nuclease-free water, gently mixed by pipetting, and incubated at 37 °C for 30 min. After double-stranded DNA (dsDNA) to ssDNA digestion, the reaction was heat-inactivated at 75 °C for 10 min. ssDNA was purified using the NEB Monarch PCR and DNA Cleanup Kit according to the manufacturer’s instructions, and eluted to a final volume of 20 µL. The resulting SELEX product was used in subsequent rounds or stored at −20 °C until used. 

### 2.5. Next-Generation Sequencing of SELEX Aptamer Rounds

The ssDNA pools from SELEX rounds 1 to 8 (R1–R8) that were bound to immobilized RNase were prepared for next-generation sequencing (NGS) analysis following amplicon sequencing library preparation. In brief, PCR was used to attach 5′ Illumina adapter overhands to the SELEX forward primer (Ill-Adapter-Selex-Forward (56 bp)), 5′-TCG TCG GCA GCG TCA GAT GTG TAT AAG AGA CAG-TAG GGA AGA GAA GGA CAT ATG AT), and reverse primer (Ill-Adapter-Selex-Reverse (57 bp)), 5′-GTC TCG TGG GCT CGG AGA TGT GTA TAA GAG ACA G-TCA AGT GGT CAT GTA CTA GTC AA). PCR reactions were carried out in 50 µL reactions containing 32 µL of nuclease-free water (ThermoFisher, Waltham, MA), 10 µL of 5X PCR buffer (Bioline, London, England), 2 µL of forward and reverse primers (20 µM each), and 1 µL of MyTaq polymerase (BioLine, London, UK). A total of 5 µL of ssDNA aptamer template was added to each reaction. The PCR cycling conditions were 3 min at 95 °C, followed by 11 cycles of 15 s at 95 °C, 15 s at 55 °C, 15 s at 72 °C, and a final extension step for 2 min at 72 °C. Due to the high initial concentration of ssDNA in Round 1, only 8 cycles were used. The PCR products were checked using UV illumination on a 2% agarose gel stained with ethidium bromide. A second NGS-PCR used primers containing adapter sequences with Illumina-specific barcodes (i7 and i5) to attach the oligonucleotides to the flow cell. For the second PCR, reactions were carried out in 25 µL reaction volumes containing 1X High-Fidelity Master Mix and 1 µM NGS_PCR#2 primers (Illumina, San Diego, CA, USA). Purified first NGS-PCR product (2.5 µL) was added to each reaction and cycled at 95 °C for 30 s, followed by 6 cycles of 10 s at 95 °C, 30 s at 55 °C, 30 s at 72 °C, and a final extension step for 2 min at 72 °C. The PCR products were purified per manufacturer’s instructions using the Agencourt AMPure XP Purification System (Thermo Fisher, Waltham, MA, USA). The final DNA concentration for each product was determined using a Qubit fluorometer 4.0 with the DNA Quantification Kit (Thermo Fisher, Waltham, MA, USA), according to the manufacturer’s instructions. The eight samples (R1–R8) were mixed in an equimolar ratio to a final concentration of 4 nM, and the final NGS library was clustered at 8 pM with 10% of the 8 pM PhiX internal control added to the run. Sequencing was performed using an Illumina MiSeq platform (San Diego, CA, USA) with the MiSeq Reagent Micro Kit v2 (150 cycles) cartridge in paired-end mode. The sequencing data were demultiplexed using bcl-2fastq2 v2.20 (Illumina, San Deigo, CA, USA).

The criteria employed for the selection of aptamers from NGS included the following: (1) unique sequences present in all 8 SELEX rounds, and (2) determining unique sequences with the highest frequency among all reads in SELEX Round 8. The frequencies of the chosen aptamers in SELEX Round 8 are shown in Table 1. 

### 2.6. Evaluation of ssDNA Aptamers Using Substrate Nuclease Detection

An evaluation of RNase digestion and protection using anti-RNase aptamers was performed with the RNaseAlert Substrate Nuclease Detection System™, according to the manufacturer’s guidelines (Integrated DNA Technology, Coralville, IA, USA). Intact ssRNA does not fluoresce, but ssRNA cleavage by RNase gives rise to fluorescence that is (1) visible under UV light, and (2) quantifiable using a fluorometer. 

The assay was carried out by adding 5 µL of 10X Buffer, 45 µL of 100 µM aptamer, and RNase at a final concentration of 20 µg/mL. Following the addition of RNase, the samples were immediately vortexed, and measurements at 470 nM were taken every 30 s for 5 min using a Qubit 4 Fluorometer (Thermo Fisher, Waltham, MA, USA). Visual inspection under UV light was conducted after 1 h and on day 5.

### 2.7. Evaluation of ssDNA Anti-RNase Aptamers Using qPCR Analysis

Enzymatic digestion reactions using purified influenza A (H3N2) viral RNA in the presence of bead-RNase conjugate in the presence of anti-RNase aptamers were evaluated in a 25 µL reaction mixture consisting of 5 µL of bead-RNase conjugate or naked (control) beads, 10 µL of Xtract-Free™ medium, and 10 µL of purified vRNA (10^6^ copies/mL). For reactions containing anti-RNase aptamers, a total of 10 µL of aptamer (0.2 µM final concentration) was used in place of Xtract-Free™ medium. The samples were mixed, incubated for 15 min at 37 °C, and magnetically partitioned for 1 min, and the soluble fraction was then transferred to a new sterile 1.5 mL tube for analysis.

Quantitative reverse transcription PCR (qRT-PCR) was used to assess aptamer inhibition of RNase. The TaqPath 1-Step RT-qPCR Master Mix (ThermoFisher Scientific, Waltham, MA, USA) was utilized following manufacturer’s instructions. A universal influenza A virus assay targeting a conserved region of the matrix gene was used with a final concentration of 0.5 µM for the forward and reverse primers, and 0.1 µM for the internal hydrolysis (TaqMan) probe, as previously described [29]. Real-time detection was carried out using a QuantStudio 5 instrument (Thermo Fisher, Waltham, MA, USA). Each qRT-PCR run included duplicate positive and negative control reactions. Lower C_T_ values indicate a higher initial viral RNA concentration, with a value >40 indicating no detection. The real-time thermocycling parameters were as follows: 50 °C for 20 min (reverse-transcription), 95 °C for 2 min (hot-start), followed by 40 cycles of 95 °C for 15 s and 60 °C for 30 s.

## 3. Results

Figure 1 provides a detailed overview of the multi-step process used to isolate and identify the highest affinity anti-RNase aptamers from a starting pool of 10^15^ candidate sequences. The SELEX procedure involved eight iterative cycles of incubation, washing, and amplification, gradually enriching for aptamer sequences that bind to the target protein, RNase A. The incubation of aptamers that bind to the bead-RNase conjugate was performed in XF medium, and at an elevated temperature of 28 °C, conditions similar to those for collection of biological samples in the field (Figure 1).

**Figure 2:**
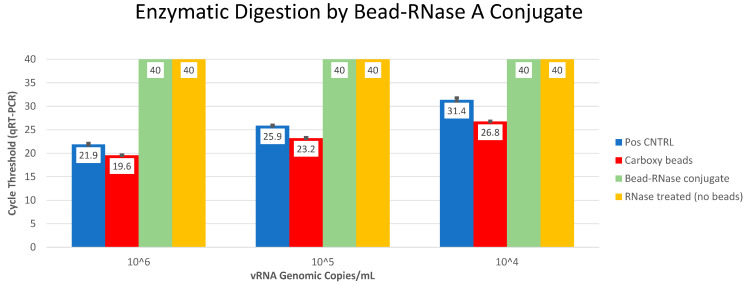
qRT-PCR results of enzymatic digestions using bead-RNase A conjugate. Pos CNTRL = reaction contains viral RNA and no RNase A (positive control); Carboxy beads = reaction contains viral RNA + carboxy beads (unbound); Bead-RNase conjugate = reaction contains viral RNA + bead-RNase conjugate. RNase A treated (no bead) = reaction contains viral RNA + equivalent RNase A (negative control). All reactions were incubated for 15 min @ 37 °C. Average real-time cycle qRT-PCR cycle threshold values from triplicate digestion reactions with standard deviation bars are shown.

Prior to SELEX, an assessment was made on chemically immobilized RNase A, i.e., paramagnetic bead RNase conjugate using qRT-PCR analysis (Figure 2). The bead-RNase conjugate was magnetically partitioned and washed three times to ensure removal of unbound enzyme. Subsequently, three concentrations (10^6^, 10^5^, and 10^4^ genomic copies/mL) of SARS-CoV-2 viral RNA were used as a substrate to confirm active enzymatic degradation by bead-immobilized RNase A. Compared to cycle threshold (C_T_) values from positive control vRNA alone (Avg C_T_ = 21.9, 25.9, and 31.4 for 10^6^, 10^5^, and 10^4^ copies/mL, respectively) and positive control vRNA incubated with unmodified carboxy beads (Avg C_T_ = 19.6, 23.2, and 26.8), equivalent vRNA from thrice-washed bead-RNase conjugate incubations revealed undetectable viral RNA (C_T_ = 40; SD = 0) for all three target concentrations. A negative control containing free RNase A and viral RNA was also included. The data clearly indicate the complete digestion of target viral RNA by the bead-RNase conjugate. The figure indicates triplicate averages with standard deviations (Figure 2).

**Figure 3:**
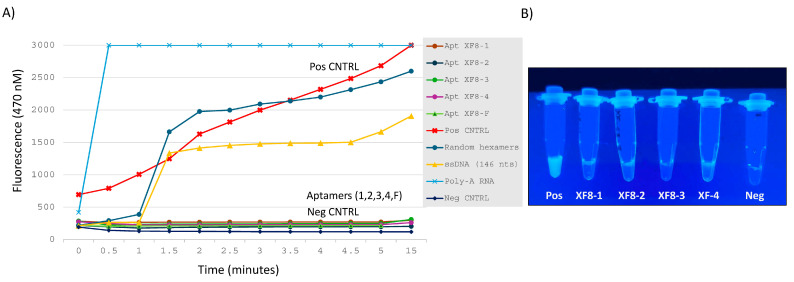
RNase A enzymatic activity using the RNaseAlert™ Substrate Nuclease Detection System (IDT, Coralville, IA, USA) from: (**A**) reactions assessed by Qubit fluorometer (Thermo Fisher, Waltham, MA, USA) measured every 30 s for 5 min and at 15 min, and (**B**) using ultraviolet light with visual inspection at 5 days. In (**A**), reactions containing each aptamer (XF8 1-4 and XF8-F) were similar to Neg CNTRL reaction. Note: In (**B**), aptamer XF8-F is not shown but was similar to aptamers XF8-1 to 4, i.e., little to no visible fluorescence compared to control.

The top five anti-RNase aptamers were chosen based on their presence in all eight SELEX rounds (as determined by next-generation sequencing) and their highest frequency (unique reads ÷ total reads in round 8 × 100) based on total reads obtained in the final eighth round (Table 1). To assess inactivation of RNase by DNA aptamers, a single-stranded RNA substrate with a 5′ fluorescein (reporter) and 3′ dark quencher was monitored fluorometrically every 30 s for 5 min, and at *t* = 15 min. After 15 minutes, ssRNA targets in the presence of the five anti-RNase aptamers exhibited minimal to no degradation, similar to controls without RNase. In contrast, reactions containing random hexamers, a ‘nonsense’ ssDNA sequence (146 nucleotide ssDNA non-aptamer), and poly-A RNA incubated with RNase A were readily degraded (Figure 3A). When examined visually under UV transillumination after 5 days, anti-RNase aptamers (XF8-1 to XF8-4) displayed minimal to no fluorescence in contrast to control reactions. This indicates that all five DNA aptamers effectively bind and inhibit RNase A, as evidenced by the absence of ssRNA digestion (Figure 3B).

**Figure 4:**
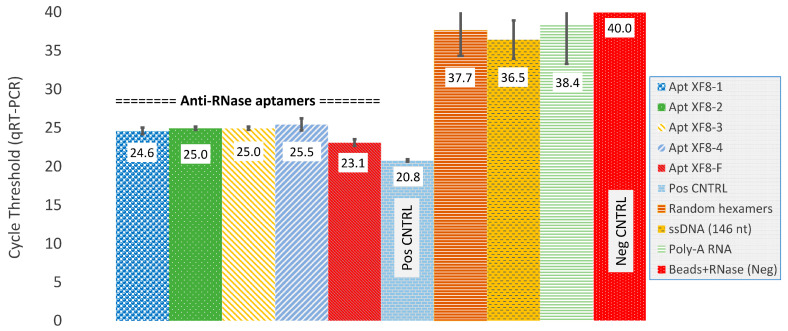
Anti-RNase aptamer protection after bead-RNase conjugate incubation as assessed by qRT-PCR. Reactions containing aptamer were similar to positive control reaction containing no bead-RNase conjugate. Random DNA hexamers, a ssDNA non-aptamer (146 bp), and poly-A RNA were readily digested and like negative control reactions containing only viral RNA plus RNase A. All reactions were incubated for 15 min @ 37 °C. Average real-time qRT-PCR cycle threshold values from triplicate reactions with standard deviation bars are shown.

Similarly, the efficacy of anti-RNase aptamers to inactivate RNase A was evaluated via qRT-PCR. This was accomplished by exposing an abundance of RNase A enzyme to naked, purified influenza A viral RNA substrate (10^6^ copies/mL) for a duration of 15 min at 37 °C. The results indicate that when compared to control reactions containing equivalent RNA concentration but no RNase A enzyme, the five aptamers exhibited comparable levels of RNase inactivation, as evidenced by the qRT-PCR cycle threshold values. Furthermore, in comparison to negative control reactions (i.e., RNA plus RNase with no aptamers), which were not detected (cycle threshold value of 40), the reactions containing aptamers exhibited cycle threshold values ranging from 14.6 to 16.9. These values translate to an 8800- to 10,200-fold reduction in RNase A activity. Conversely, other oligos such as random hexamers, a 146-nucleotide ssDNA oligomer, and poly-A RNA did not indicate any significant inactivation of RNase A (Figure 4).

## 4. Discussion

RNase A is a small enzyme (13.7 kilodaltons) consisting of a single 124 amino acid polypeptide chain. The active site of RNase A contains highly conserved histidine, lysine, and tyrosine residues that form hydrogen bonds and electrostatic interactions with the phosphate backbone of RNA, enabling the enzyme to cleave phosphodiester bonds of RNA into component nucleotides [11,12,13,14,15]. The enzyme predominantly targets single-stranded RNA regions rich in cytosine and uracil residues. RNases are particularly problematic in RNA detection and genomics applications, since they are highly resistant to heat degradation. Additionally, RNases are abundant in secretions and skin as well as in copious amounts in the cells of collected biospecimens.

In a typical molecular diagnostic laboratory, special precautions are commonly used to minimize the deleterious effects of ubiquitous contamination by RNases in reagents and kits used for genomics application. Some molecular procedures that involve RNA analysis employ the use of an RNase inhibitor (RI), natural or recombinant proteins that bind to and inhibit RNase enzymes. RNase inhibitors are often used in kits for isolating RNA from collected specimens. During the nucleic acid extraction and isolation process, cellular membranes are disrupted, releasing endogenous RNases that rapidly degrade target RNA. Adding an RI to RNA extraction buffers or other genomic steps prevents this degradation, enabling the integrity of high-quality, intact RNA for downstream applications including RT-PCR, RNA-Seq, and transcriptome analysis. However, the use of RIs in sample collection mediums presents several insurmountable challenges. RNase inhibitors are relatively expensive reagents, thus an effective concentration ensuring complete inhibition is cost prohibitive, particularly in a collection medium where liquid volumes are usually 1 to 3 mL per vial. Furthermore, commercially available RIs (i.e., recombinant enzymes) present signification stability challenges over extended shelf lives and temperature ranges.

Sample collection is the first and most critical step in molecular diagnostic testing. Thus, the appropriate transport medium is essential for maintaining the integrity of genomic targets. First-generation transport medium, known commonly as viral and universal transport media (VTM/UTM), were developed in the 1980s for the purpose of maintaining microbial viability for culture detection techniques. Commercial VTM/UTM typically consist of identical formulations that include balanced salts, antibiotics to prevent bacterial and fungal growth, a buffer and indicator to maintain pH, and bovine serum albumin to support virus survival. In 2007, second-generation media, referred to commonly as molecular transport medium (MTM), was developed to shear and disrupt cellular membranes for subsequent nucleic acid testing. Second-generation transport mediums typically contain guanidine, e.g., guanidine thiocyanate and one or more detergents, e.g., *N*-lauroylsarcosine designed for chemical disruption of cellular lipid bilayers and stabilization of nucleic acids, making MTM suitable for use only in nucleic acid tests such as PCR [21].

Despite their continued use, both VTM/UTM and MTM have limitations. One drawback of VTM/UTM is that they can only preserve live viruses for finite periods, i.e., typically 48–72 h when kept refrigerated, after which the viability of the virus significantly decreases, leading to possible false negative results [30,31,32,33]. Furthermore, most VTM/UTM usually require cold-chain transportation, and collected pathogens are potentially infectious, adding complexity and cost to sample handling, shipment, and transport [30,31,32,33]. Conversely, MTM contains harsh and hazardous chemicals, requires a nucleic acid extraction step, and does not maintain viral infectivity and protein structure, which is essential for virological studies, culture-based detection methods, and rapid antigen/lateral flow tests.

The utilization of aptamers for the purpose of binding and inactivating RNases from collected samples represents a novel and innovative approach in sample collection. This method allows for the preservation of RNA integrity at ambient or elevated temperatures, eliminating the need for cold-chain transportation, and contributing to a safer and more environmentally friendly medium for point-of-care collection and shipping. Our study entailed eight rounds of SELEX selecting for anti-RNase aptamers (Figure 1, Table 1), and was achieved using paramagnetic bead-RNase conjugate (Figure 2). Subsequently, we demonstrated binding and inhibition of bead-RNase conjugate by anti-RNase aptamers in Xtract-Free™ collection medium using direct enzymatic fluorometer assessment (Figure 3) and real-time qRT-PCR (Figure 4). Integration of harmless DNA aptamers into a chemically safe collection medium, i.e., XF, significantly improves preservation of collected samples, especially in remote or field-based collection scenarios where rapid processing is not feasible.

In contrast to other mediums containing harsh chaotropic and surfactant agents for chemically inactivating nucleases, a DNA aptamer consists solely of a short and unique sequence of nucleic acid that has no significant impact on downstream applications, including direct use of extraction-free PCR. Furthermore, the use of ‘carrier RNA’ during nucleic acid extraction has been demonstrated to improve the efficiency and yield of RNA and DNA [34]. Nucleic acid carrier, utilized in some commercial MTMs and extraction kits, increases the extraction efficiency from low target samples during nucleic acid extraction [34]. Including DNA aptamers in collection medium to enhance RNA preservation by inactivation of RNase may also contribute to improving the efficiency and yield of nucleic acids by the aptamer functioning as a carrier for low target sample RNA during nucleic acid extraction.

The incorporation of DNA aptamers into Xtract-Free™, a chemically safe and environmentally friendly ‘third generation’ collection medium, is a significant advancement in preserving RNA integrity. An anti-RNase aptamer, consisting of merely a short single-stranded DNA sequence, provides a cost-effective and more user-friendly alternative to sample collection media such as MTMs that contain hazardous and environmentally harmful chemicals for inactivating RNase. The targeting and inactivation of RNase by DNA aptamers was shown to effectively protect RNA, thereby improving the detection of RNA genomic targets.

## 5. Conclusions

This report introduces the first ever use of anti-RNase aptamers selected specifically to target and neutralize RNase A. Xtract-Free™ collection medium, which incorporates these aptamers, offers a safe and environmentally friendly option for molecular and lateral flow diagnostic tests, eliminating the need for nucleic acid extraction prior to PCR. Inclusion of anti-RNase aptamer in collection and transport mediums ensures preservation and stabilization of RNA for PCR and other RNA-related genomic applications, e.g., RNA-Seq and transcriptome analysis. Xtract-Free™ medium, containing anti-RNA aptamers, expands the scope of collection mediums beyond conventional VTM/UTM and MTM to meet a growing need for safe mediums to use in point-of-care, self-collection, return mail, and home collection formats.

## Figures and Tables

**Table 1:** Characterization of anti-RNase aptamers.

			RNase Inhibition According to qRT-PCR		
Seq ID	(N40) Aptamer Sequence (5′-3′ Orientation) ^1^	Rd8 NGS Reads (% Total Reads) ^2^	Cycle Threshold Difference ^3^	Log(10) Reduction ^4^	Enzymatic Inhibition (Fold-Reduction) ^5^
Apt XF8-1	GCTGCCGACGATTGACTAGTACATGACCACTTGACTGTCT	114 (0.64%)	15.4	4.7	9400
Apt XF8-2	AGGGTGAGCATCCGCATAACAATAGTGCTGTTTAGTTGG	58 (0.33%)	15.1	4.6	9200
Apt XF8-3	CTAACTTGAATAAATACCAGCACCAGACTGCCCGCGTTT	35 (0.20%)	15.1	4.6	9200
Apt XF8-4	GGACGGGTATACACTAGACAACAACAAGGAACACTCTTTC	21 (0.12%)	14.6	4.4	8800
Apt XF8-F	GCTTTCCATGTCGTTATCCTAGGGGCTGTTAGCTAATTTC	18 (0.1%)	16.9	5.1	10,200

^1^ The 40 nucleotide variable aptamer sequence. ^2^ The total number of next-generation sequencing reads in SELEX round 8. Percent (%) total = reads ÷ total reads (17,803 total reads) × 100. ^3^ Difference in cycle threshold values from negative control reactions containing beads-RNase conjugate + vRNA (no aptamer). ^4^ Log reduction in RNase A acitivity where 1 log(10) = 3.3 qRT-PCR cycle threshold values. ^5^ The X-fold RNase A inhibition, e.g., aptamer XF8-1 is 4.7 logs ÷ 5 logs × 10,000 (5 logs) = 9400-fold difference vs. negative CNTL containing equivalent bead-RNase conjugate and viral RNA targets with no aptamer.

## Data Availability

The data pertaining the those presented in the Figures is available upon request.
